# Choking under the pressure of competition: A complete statistical investigation of pressure kicks in the NFL, 2000–2017

**DOI:** 10.1371/journal.pone.0214096

**Published:** 2019-04-02

**Authors:** Nai-Wei Hsu, Kai-Shuo Liu, Shun-Chuan Chang

**Affiliations:** 1 Department of Medicine, Mackay Medical College, New Taipei City, Taiwan; 2 Holistic Education Center, Mackay Medical College, New Taipei City, Taiwan; Texas A&M University, UNITED STATES

## Abstract

In the NFL, kickers play a special role in determining the outcome of a match. There is a significant body of literature attributing the success of kicks to observed environmental and situational factors. However, the significance of these is not subject to agreement. In this study, we synthesize the deterministic and stochastic models based on data from the 2000–2017 NFL seasons to identify significant conditions associated with “choking.” This study’s empirical findings focus on integrating the statistical evidence on causality of skill and performance, and the interpretation of observed and unobserved heterogeneity of kicks, on the intervention effect of the new extra-point rule in the NFL since 2015, and on providing an in-depth evaluation of the impact of competition pressure.

## Introduction

Sports performance under the pressure of competition can produce notable changes when athletes are under increased pressure at a critical moment or during a particularly important match. Among such changes is “choking,” which has been extensively discussed in previous literature. The term “choking” is used when athletes do not perform up to their usual standard under pressure, which means poorer performance or functionality [1–3]; it can be regarded as a severe problem for professional players because good or bad performances at critical moments result in great achievements or damage their reputation in addition to monetary gains or losses [4–6]. A pressure kick is arguably one of the most competitive tasks an American football field goal kicker experiences because its pressure is a potentially game-winning situation [7]. For example, the National Football League’s (NFL) championship match—the Super Bowl—remains a sports event that is highly anticipated worldwide. In the 1991 Super Bowl, Buffalo Bills kicker Scott Norwood missed a field goal during the final minute, which led to his team’s defeat by one point. As a result, his career as a professional footballer was doomed. Furthermore, the kick he missed lives on in posterity in the historical record of the NFL. This intriguing game begs the question: does the statistical evidence really suggest that in some circumstances NFL kickers are prone to choking?

In the vast majority of field goal attempts and all kickoffs, credit or blame for the outcome can be attributed fully to the kicker [8]. On November 26, 2016, another important event took place in 10 of the NFL regular matches that day. Ten kickers together created an incredible record of the highest number of point after touchdown (PAT; extra points) failures happening in a single day, totaling 12. There were only 8 PAT failures the entire 2014 season, but on November 26, 2016, the failures in a single day exceeded those of the entire 2014 season. The reasons behind such failures are also the motivations behind our research; the main reason possibly being changes to the rules of the tournament. A new rule introduced in 2015 moved the line of scrimmage for a PAT attempt from its original 2-yard line to the newly set 15-yard line (lengthening PAT attempts from 20 to 33 yards). Although the PAT attempt from the fixed 15-yard line of scrimmage was not far for professional kickers, based on the official NFL statistics, these extended yards reduced the success rate of PAT attempts from 99% to 93%–94% after 2015 NFL season. Traditional measurements for a kicker’s contribution include the percentage of field goals made or points scored (a team gains three points for a field goal and one for a PAT) [8]. The 2015 PAT rule change may result in more uncertainty for game outcomes and encourage football teams to select more skillful kickers to mitigate against losses in matches with a small points’ gap.

### Suboptimal performance at critical moments in sports games: The phenomenon of “choking” under pressure

“Choking” is a term that originated from English medical terminology, which mainly describes the physiological phenomenon of sudden suffocation. Sports psychologists say the term can be defined as the phenomenon in which a visible decay occurs in the process of regular motor execution under psychological pressure [9]. At present, there are two main theoretical hypotheses to the mechanism that causes choking in athletes: one is the “distraction hypothesis” proposed by a sports psychologist [10, 11], and the other one is the “automatic execution hypothesis” proposed by social psychologists [2, 12, 13]. However, with most game records in practical applications, one cannot really address what is likely transpiring internally within the performer and thus inference is largely futile. For example, situational pressure can cause an increase in distraction as crowds can become potentially too noisy, which can lead to the athlete’s poor performance, rather than an inhibition of “automatic execution”.

The above two hypotheses can be said to be *sui generis*. Nideffer [11] suggested that choking was a result of an athlete’s attention turning excessively toward introspection (information unrelated to that sport itself). Nideffer and Sagal [14] argue that in a match, when an athlete became aware of the importance of the match (attention turning toward introspection), there would be an increase in sports anxiety, which in turn generated physiological stress, including increased heartbeats, rises in blood pressure, and muscle tension. These physiological responses would distract the attention of athletes, who eventually ended up choking. One the other hand, the automatic execution hypothesis proposes that an individual often has to pay too much attention to the execution details of motion. Although choking is used when athletes do not perform up to their usual standard, some elite athletes can still exhibit mental toughness and use avoidance coping in a high-pressure situation to focus on the task [15, 16].

It is common for choking to be found in many sports games, yet empirical NFL data conducted regarding choking research remains relatively unexplored. Although psychologists and interdisciplinary researchers have been researching choking for decades, sports analytics has mainly focused choking research on examining the performance of free throws in basketball [17–19] and on golf [20, 21]. In general, the conclusions of these studies differ widely, with some discovering evidence of choking while others finding no significant influence. In the past few years, behavioral economists have also begun to study sports performance under competition pressure, which was mainly done through experiments [22] or sports data analysis [23–25]. Experiments allow people to control external factors and the manipulation of the causes of choking in lab or field experiments has been shown to stimulate competition pressure effectively. However, in the context of actual professional matches, there are always other factors, such as timing, location, and people’s support, including, but not limited to, immense attention and social facilitation, which the experimental method cannot all adequately illustrate. However, in the face of emerging technological advances in the contemporary world, producing a detailed play-by-play type of real data for sports analytics has become possible.

### How statistical models interpret NFL kickers’ performance

Peter Drucker [26] gave an interpretation of the functions of statistical models in his book, *The Age of Discontinuity*: “All we can ever predict is continuity that extends yesterday’s trends into tomorrow. What has already happened is the only thing we can project and the only thing that can be quantified. But these continuing trends, however important, are only one dimension of the future.” Thus, the construction and functions of statistical models are often used to predict or explain phenomena. By extension, like the common causal inference that employs the regression model to predict kickers’ “field goal success rate,” it involves the use of many observable influencing factors. However, those involving unobserved heterogeneity such as the hidden trait to anti-stress, true ability to score, or other non-attributive dimensions of players remain subtle.

On the one special literature with application to stochastic model, Morrison and Kalwani [27] employed a probability distribution model to question whether it is skill or luck that caused some kickers to appear to perform better. To accurately capture the heterogeneity of the actual ability of the players, they used the beta distribution of statistical probability to describe it, and succeeded in including the heterogeneity of the players’ ability in the beta-binomial model of success or failure in field goals for consideration. The research confirmed that even when faced with the condition of different kicking distances, there was indeed no significant difference in the actual ability among the kickers across all NFL teams. The conclusion may totally neglect the unique role of NFL kickers.

However, in mainstream literature there have been significant studies that emphasized investigating possible factors that may affect the kick success rate, and which explored the importance of each factor using logistic regression models. The following is a collation of the results of several significant studies.

The first thesis concerning kickers was published in 1985 [28]; it employed logistic analysis in investigating the probability of a kicker’s success or failure in field goals. In 1998, as the play-by-play of the games became increasingly detailed, Bilder and Loughin [29] established a more comprehensive method of logistic analysis. Berry and Wood [30] proposed yet another model, with factors including kicking distance and nine binary variables. However, it differed from the 1998 study in that it proposed that these three factors—an indoor stadium, precipitation, and natural turf—would affect the kick success rate. Goldschmeid et al. [7] used data from six seasons in the hope of further observing the influence of high-pressure situations on the kick success rate. A pressure kick in this study was defined as one that was performed within one minute or less of the end of regulation time when the kicking team was behind by three points or less (ties included), or during overtime, and the success or failure of the kick would determine whether a team could tie the game or would place them in the lead to win the match. Finally, using hierarchical regression, and controlling for kicking distance, they discovered that the icing strategy, whereby the opposing team would call a timeout before the kick, could indeed reduce the kick success rate of kickers. That is to say, icing, interrupting an individual just prior to a task may lead to damaging effects or decreased performance [31].

Clark et al. [32] have made significant progress concerning kick statistics. They made use of the massive kicks data across 12 seasons from 2000 to 2011 to study the variables related to the kick success rate. With distance as the primary variable, they carried out their research using six environmental conditions and four situational/psychological factors, namely high and low temperature (with 50 degrees Fahrenheit as the dividing line); altitude (especially with reference to Denver home games); turf of stadium (artificial or natural); precipitation; wind speed (with 10 mph as the dividing line); and humidity (with the relative humidity of 60% as the dividing line) for the former and post-season vs. regular season, pressure situations, home vs. away, and an “icing” time-out before the kick (as defined in Table 1) for the latter. Except for humidity, the other environmental conditions attained significant differences in the success rate. However, the study found that none of the situational or psychological factors affected the kick success rate. Pasteur and Cunningham-Rhoads [33] utilized the data across three seasons (2008–2011) to construct a model similar to Clark et al.’s [32] study. Their primary difference lay in that the former’s data contained “continuous” weather variables. However, the study by Clark et al. [32] utilized extensive data and was generally recognized as a credible study, which also means that temperature, altitude, precipitation, and nature of turf would have influences on kickers. Nevertheless, the conclusions from this study still generated many questions [8]: for example, does the observed temperature dependence reflect only a negative impact of cold conditions, or are hot conditions beneficial? Does the rate or type (rain versus snow) of precipitation during the game matter? Is the turf quality of a natural grass field different early in the season than in a December or January game, to the point that it affects kicking success? What is more, we seriously suspected why none of four other situational/psychological factors was found to be significant.

**Table 1:** Detailed definitions of variables.

Variable		Data base	Research	Special definition (S1, S2 and S3 Figs)
Distance		Yards	The distance between goal kicker which is measured by yard.	-
Environmental	Cold temperature	°F	Equals 1 if temperature is less than 50°F, and 0 otherwise.	-6 ~ 45°F = lower 46 ~ 56°F = low 57 ~ 65°F = medium 66 ~ 73°F = high 74 ~ 109°F = higher
	Field surface	A Turf Titan, MomentumTurf, Artificial Turf, NeXTurf, AstroPlay, Sportex, AstroTurf, SportGrass, DD GrassMaster, and UBU Speed Series S5M = 1, and Grass = 0.	Equals 1 if it is artificial grass, and 0 for natural grass.	1–6 week: Early season. 7–12 week: Mid season. >13 week: Late season.
	Altitude	Estadio Azteca, Invesco Field at Mile High, Mile High stadium, and Sports Authority Field at Mile High = 1, and other stadium = 0.	Equals 1 if stadium higher than 4000ft, and 0 otherwise.	-
	Precipitation	Chance rain, Clear, Closed roof, Cloudy, Cold, Covered roof, Dome, Fair, Foggy, Hazy, Mostly cloudy, Mostly sunny, Overcast, Partly cloudy, Partly sunny, Sunny, Thunderstorms, and Windy = 1, and Flurries, Light rain, Light showers, Light snow, Rain, Showers, and Snow = 1.	Equals 1 if it is raining or snowing, and 0 otherwise.	Light rain: light shower and light rain. Light snow: light flurries and snow. Rain: rain and shower. Snow: snow
	Windy	Equals 1 if wind speed is higher than 10 mph, and 0 otherwise.		-
	Humidity	Equals 1 if humidity is higher than 60%, and 0 otherwise.		-
Situational	Postseason	Equals 1 if week is more than 17, and 0 otherwise.		-
	Away	Equals 1 if offensive team is not the home team, and 0 otherwise.		-
	Icing kicker	-	Equals 1 if a timeout is called by either head coach, and 0 otherwise.	-
	Icing kicker offensive	-	Equals 1 if a timeout is called by offensive head coach, and 0 otherwise	-
	Icing kicker defensive	-	Equals 1 if a timeout is called by defensive head coach, and 0 otherwise	-
	High situational pressure	-	S1 Table	-
	Extra point pressure	-	S2 Table	-

We know from the discussion above that literature examining the behaviors of NFL kickers from the perspective of probability theory is relatively lacking. In contrast, if one wholly assumes determinism and exhaustively lists all explanatory factors, which believes that the details of each kick will be well-known, this then might lead to overlooking the unrecorded, unobserved, unexpected, but actual situations in the real world. In fact, there are logical and practical difficulties in explaining the whole causal relationship between the performances of NFL kickers, and predicting the robust occurrence of successes or failures in light of principles of determinism. In more explicit terms, the residuals in multiple regression analysis are often used where the knowledge of other factors is lacking, or errors exist in measurements [34]; the probability connotation of residuals is then used to interpret the contingency structure in our research models.

## Materials and methods

### Research data

The data the NFL publishes on its website usually concerns season-by-season data on players in relation to their positions, such as the total passing yards of the quarterbacks and the field goal percentage (FG%) of the kickers, but the circumstantial details of the matches are often missing. Information cited in this research, such as temperature, wind speed, field environment, pressure faced by the players, and offensive and defensive strategies at crucial moments of a match, is all difficult to come by in the NFL published open data.

The statistician Maksim Horowitz of Carnegie Mellon University created an R package (nflscrapR) using the play-by-play details provided by NFL’s API. Moreover, ArmchairAnalysis.com (https://www.armchairanalysis.com/) that we utilized in this study (also in Clark et al. [32]), is one of the paid websites offering play-by-play data, and has much more detailed match data spanning 18 years, from 2000 to 2017. Its annually updated database contains not only the relevant environmental and circumstantial information on the day of each match but also detailed information of the player performances on the offensive and defensive teams.

### Statistical analysis

#### Study 1: Conventional logistic regression model and residual analysis

We first conducted logistic regression analyses to confirm the factors affecting FG and PAT according to different time span and PAT rule changes. The dependent variable for the logistic regression in (1) below is binary; “1” denotes “scored,” “0” denotes “missed.” The logistic regression model is useful as the common aim to find out the statistical relations between the binary dependent variable and a set of independent variables (categorical or continuous). The independent variables in our study include environmental and situational factors, especially some pre-determined stress conditions mentioned on the previous study [32]. The model is as follows:

$$\ln(\frac{p}{1-p})=\beta_{0}+\beta_{1}X_{1}+\beta_{2}X_{2}+\beta_{3}X_{3}+\ldots +\beta_{k}X_{k}+\varepsilon$$ (1)

where X_i_ is the corresponding independent (environmental or situational) variable, β_i_ is the estimated parameter. These coefficients β_i_ are used to calculate the probability (*p*) that a particular FG or PAT will be successful.

Second, we then combined only the significant environmental factors from all FG or PAT data separately into two different adjusted-effect models that take into account the distances, weather conditions, etc. of the kickers’ attempts. For instance, using the residual value subtracts the model’s predicted likelihood from the actual outcome of a FG (1 for a make, 0 for a miss) as a new derived variable, named as a kicker’s true performance. We can drill it down into the situational perspective on the kicker’s performance under the pressure of competition. As one example, kickers with residual values out of a predicted model of FG are providing additional points (AP), sum of (3* residuals) beyond what would be expected of an average kicker given the same opportunities [32]. We can also define a new metric—extremely great play by one kicker, equal to the summation of one kicker’s residuals under the model’s predicted likelihood of success (e.g., less than 20 percentiles from all population, P_20_) divided by counts of these attempts.

#### Study 2: Logistic quantile regression

We further conducted logistic quantile regression [35, 36] that can be considered a comprehensive approach to inference about the conditional distribution of bounded outcomes (the derived variable via adjusting for the effects from the Study 1 in our statistical analysis) given a set of aforementioned situational factors. It allows a deeper understanding than the mean regression methods. The estimates of all the regression coefficients with different quantiles represent the whole trend variation, and especially reveal a full view about the influences of competition pressure.

#### Study 3: Beta-binomial model

Unobserved heterogeneity among the kick behaviors of individual players lingered beside a core premise of how differentiated NFL kickers are. Morrison and Kalwani [27] applied the beta-binomial model to show that the strength of NFL kickers did not show any statistically significant variation. However, what about after the extra-point rule change, and when facing different pressure situations? In the following steps, we verified the method and classification of kicking distances adopted by Morrison and Kalwani [27] to present the NFL kicker’s long-range performance for FG and PAT, from 2000 to 2017.

1. FG or PAT success expressed in binomial distribution.

For instance, assuming that the FG samples come from *k* players (*i* = 1, 2, 3,…,k). If the total number of FG attempts of player *i* is set as, the FG% for player is, and is the total number of field goals of that player, then has the characteristic of binomial distribution, and its mean and variance are as follows:

$$P(X_{i}=x|P_{i},M_{i})=\left(\begin{array}{l} M_{i}\\ x \end{array}\right)P_{i}^{x}{\left(1-P_{i}\right)}^{M_{i}-x},x=0,1,2,\ldots ,M_{i}$$ (2)

$$E\left(x_{i}|P_{i},M_{i}\right)=P_{i}M_{i},Var\left(x_{i}|P_{i},M_{i}\right)=P_{i}(1-P_{i})M_{i}.$$ (3)

Both simple FGs and PAT attempts can be calculated separately.

2. Heterogeneity in ability of different kickers expressed in beta distribution.

Due to the difference between players, we hypothesize that every player *i* has a different *P*_*i*_. In other words, *P*_*i*_ stems from a particular distribution, and beta distribution is one of the suitable distribution models whose range of values is between 0 and 1, and this is appropriate for describing FG percentage *P*_*i*_. The principle is as shown in Formula (4) below, where *B*(*α*,*β*) is the beta function [37], and *P*_*i*_ carries the characteristics of beta distribution across all kickers *i*, their mean and variance shown in Formula (5) below:

$$\begin{array}{l} g\left(P_{i}|\alpha ,\beta \right)=\frac{P_{i}^{\alpha -1}{\left(1-P_{i}\right)}^{\beta -1}}{B(\alpha ,\beta )},0<P_{i}<1,\\ where\;B(\alpha ,\beta )=\int_{0}^{1}t^{\alpha -1}{\left(1-t\right)}^{\beta -1}dt; \end{array}$$ (4)

$$\begin{array}{l} E(P_{i}|\alpha ,\beta )=\frac{\alpha }{\alpha +\beta },\\ Var\left(P_{i}|\alpha ,\beta \right)=\frac{\alpha \beta }{{\left(\alpha +\beta \right)}^{2}(\alpha +\beta +1)}. \end{array}$$ (5)

We adopted the approach by Morrison and Kalwani [27], re-setting the polarization parameter, φ = 1/(α + β + 1) to examine the differences in kicker performance. The variance in beta distribution (from Formula (5)) can also be expressed with the same parameter: Variance = αβ/[(α + β)^2∙ (α + β + 1)] = [1/(α + β + 1)]∙[α/(α + β)]∙[β/(α + β)] = φ∙μ∙(1-μ). When μ is a fixed value in the data, only φ affects the variance, and both are directly proportional. Therefore, a greater polarization parameter implies a greater variance in the group of kickers, reflecting the different spread in strength among them.

#### Study 4: Incorporating covariates in beta-binomial model

Finally, we tried to link the beta-binomial model above to observe the changes in the mean or variance in Formula (5) in relation to the FGs, PAT, and other aspects under different distances to get an idea of average strength and whether there is heterogeneity in the distribution of strength between kickers. Then the following beta regression models adopted by Simas et al. [38] will be used in our study, more specifically for every player, y_i_ ~ B(μ_i_, φ_i_) independently, *i* = 1, … *n*, and as follows:

$$g_{1}(\mu_{i})=x_{i}^{T}\beta =\sum k\beta_{j}x_{ik},$$ (6)

$$g_{2}(\phi_{i})=z_{i}^{T}\gamma =\sum h\gamma_{h}z_{ih}.$$ (7)

where β = (β_1_, … β_k_)^⊤^, γ = (γ_1_, … γ_h_)^⊤^, k+h < n, are the sets of regression coefficients in the two equations, and x_i_ and z_i_ are covariates. We can compare the central tendency and dispersion level of the differences in strength of kickers to see if they are related to any observable variable in Formulas (6) and (7), such as a kickers’ extremely great play, added points metric, or the susceptibility to stress derived from kickers’ true performance (in our study, defining the summation of residuals under high pressure over attempts as susceptibility to stress individually). We can then identify which variable can account for the variations of strength among different kickers, and eventually achieve an explanation as to whether the kicker effect with different skill levels or stress resistance really exists or not.

In the next results section, the data is analyzed in a number of different modes above, such as logistic regression, logistic quantile regression, beta-binomial model, and variable dispersion beta regression model to describe observed and unobserved statistical heterogeneity of kicks. To empirically expand looking at the interactive dimensions of these significant conditions regarding pressure kicks, a combination of *R* (version 3.5.0) and *R* packages, including logit, lqr, betareg, and tidyverse, were used for statistical model construction and analysis.

## Results

First of all, we divided the NFL kicks data into two parts: FG and PAT (extra points). All 12,389 FG and 15,871 PAT attempts included in our research were from the 2000–2017 NFL play-by-play dataset of ArmchairAnalysis.com, which had been used in the previous study for 12 seasons from 2000 to 2011 [32]. For every FG attempt, the distance was identified along with the values of environmental (temperature, field surface, altitude, precipitation, wind speed, and humidity) and situational (regular season vs. post-season, situational pressure, home vs. away, and “icing”) explanatory variables; the same as those in the previous study [32]. Most raw continuous explanatory variables (e.g., temperature in °F) in the database were also converted into reasonable categorical variables. Details and justification for these definitions of variables can be found in S1 and S2 Tables.

### Comparing observed heterogeneity of kicks explained by the previous baseline study

We apply logistic regression on the same variables proposed by Clark et al. [32], and the results are indicated in Table 2. As seen in Table 2, from 2000 to 2010, similar to the results of Table 1: Comprehensive Logistic Regression Model (2000 to 2011) in the study [32], the model coefficients also show that longer kicks, cold temperatures, precipitation, and high winds reduce success rates of a made FG, while kicking on turf and at altitude improve the likelihood (all *p* < 0.05). None of the situational pressure or psychological factors can have a significant impact on the kick outcome.

**Table 2:** Logistic regression on environmental and situational variables.

**variable**		**Field Goal**			**Point After Touchdown**		
		(1)2000-2010	(2)2011-2017	(3)2000-2017	(4)2000-2014	(5)2015-2017	(6)2000-2017
	Intercept	5.70(0.17)***	6.23(0.23)***	5.77(0.13)***	4.91(0.21)***	3.06(0.23)***	7.54(0.28)***
	Distance	-0.10(0.00)***	-0.10(0.00)***	-0.10(0.00)***	-	-	-0.13(0.00)***
Environmental	Temperature	-0.30(0.07)***	-0.31(0.09)**	-0.30(0.05)***	-0.39(0.17)*	-0.07(0.18)	-0.25(0.12)*
	Turf	0.21(0.07)**	0.15(0.09)	0.24(0.05)***	0.61(0.20)**	0.00(0.17)	0.27(0.13)*
	Altitude	0.72(0.17)***	-0.01(0.19)	0.46(0.13)***	-0.22(0.34)	0.83(0.59)	0.11(0.29)
	Precipitation	-0.23(0.11)*	-0.49(0.16)**	-0.34(0.09)***	-0.28(0.27)	-0.59(0.27)*	-0.39(0.19)*
	Wind speed	-0.14(0.06)*	-0.21(0.08)*	-0.19(0.05)***	-0.24(0.16)	-0.23(0.16)	-0.22(0.11)†
	Humidity	0.00(0.06)	-0.09(0.08)	-0.04(0.05)	0.10(0.17)	-0.06(0.17)	-0.09(0.12)
Situational	Post-season	-0.09(0.15)	0.71(0.26)**	0.11(0.13)	0.74(0.51)	-0.21(0.41)	0.26(0.31)
	Pressure	0.00(0.13)	-0.10(0.18)	-0.06(0.11)	-0.36(0.11)**	-0.19(0.16)	-0.27(0.09)**
	Away	0.02(0.06)	-0.03(0.08)	0.01(0.04)	0.02(0.16)	-0.01(0.16)	0.01(0.11)
	Icing	-0.14(0.09)	-0.20(0.12) †	-0.15(0.07)*	-0.96(0.42)*	12.66(428.74)	-0.77(0.43) †

Here

*** refers to “*p*-value” less than 0.001

** less than 0.01

* less than 0.05, and

† less than 0.1.

All reported cells were presented as coefficient (SE). SE: standard error. A timeout called by either head coach was considered “icing the kicker,” the same way by which Clark et al. [32] analyzed the icing strategy. Categorizing “icing” as either a timeout called by the opposing coach (timeout mostly by the rivals), or timeout called by one’s own coach had a negative effect as well, but not significant. These are the coefficient values for the logistic regression equation for predicting the dependent variable from the independent variable. They are in log-odds units.

Validating the robustness of this popular regression method, we find a relationship between post-season, icing strategy, and a made FG in columns 2 and 3 that does not concur with previous reports from 2000–2010. From 2011–2017, column 2 shows that factors with significant impact include distance, low temperature, precipitation, it being windy, and whether or not it is the playoffs (*p* < 0.05). Upon comparing, it can be seen that the major difference between all 2000–2017 NFL seasons and the 2000–2010 is the “icing the kicker” effect shown in columns 1 and 3, meaning that a timeout called by either head coach before kicking still has some negative impact in terms of skirting the boundary of different significance levels (at *p* = 0.10 or *p* = 0.05) on FG percentage according to sampling from a different time duration. For instance, the coefficient (or parameter estimate) for the variable icing in column 3 is -1.05, this means that for a one-unit increase in the (0, 1) codes of icing (in other words, going from no timeout to called timeout), we expect a 1.05 decrease in the log-odds of successful performance for FG, holding all other independent variables constant.

Column 4 in Table 2 shows the results from 2000–2014 and that the factors with significant impact (*p* < 0.05) on PAT conversion are temperatures, whether the field surface is artificial turf, situational pressure (7 levels as a ordinal variable in S2 Table), and icing the kicker; while column 5 in Table 2 shows the results from 2015–2017 and that those factors with significant impact on PAT conversion in column 4 after implementing the new extra-point rule cease to be significant. In particular, column 6 in Table 2 shows the results from 2000–2017 and that factors with significant impact (*p* < 0.05) are distance, cold, field surface, precipitation, and situational pressure. Wind and icing the kicker are significant at the 10% level. The greater the distance, the lower the conversion rate; this also reflects the effect of the extra-point rule change in the 2015 season.

We can see from Table 2 that kickers are susceptible not only to environmental factors, but also to situational pressure or psychological factors especially when attempting PAT conversion before 2015. In our additional 2000–2017 study which included temperature, type of precipitation, and turf quality change with time, we can try to answer the questions [8] raised earlier. For instance, when using more specific categories of variable instead of binary (shown in Table 1), we find that temperature and type of precipitation have a nonlinear relationship versus FG percentage, with snow and decreasing temperature, in general, associated with lower FG percentage (S1 and S3 Figs). Nonlinearity of factor effect, especially lowest FG percentage, can be primarily attributed to the second low level of temperature and the light snow type. In addition, the turf quality of a natural grass field shows a greater non-differential pattern on kicking success earlier in the season than in a December or January game (S2 Fig).

### Kickers’ true performance (after adjusting for the distances, environmental factors) vs. independent situational factors

The dependent variable of logistic quantile regression model in Table 3 were the residuals (actual outcome of FG—model’s predicted likelihood of success) firstly estimated with all the significant variables (except for icing) derived from column 3 of Table 2. The probability of each particular kick after adjusting for the difficulty of their kicking attempts given the specific outward environmental influences and distances could be estimated as a new outcome variable which allows us to represent kickers’ true performance. Table 3 reports the estimated coefficients and standard errors for testing statistically significant variables at the 5th, 10th, 20th, 90th, and 95th percentile of its distribution. Consequently, it presents a broader view of the situational variables connected with this new two-directional metric of kickers’ true performance. The greater the positive value of kickers’ true performance rate, the greater the ability to conquer obstacle challenge from environmental influences and distance. On the other hand, the greater the negative value of kickers’ true performance rate, the greater the occurrence of missed kicks even under good environmental conditions and short distances.

**Table 3:** Logistic quantile regression model of field goal (2000–2017).

Variable	Quantile				
	0.05	0.1	0.2	0.9	0.95
Intercept (*β*_1_)	-2.41(+0.01)***	-1.85(+0.02)***	0.09(+0.01)***	0.67(+0.00)***	0.81(+0.00)***
Postseason (*β*_2_)	-0.01(+0.05)	-0.04(+0.06)	0.00(+0.03)	0.02(+0.02)	0.01(+0.02)
Away (*β*_3_)	-0.08(+0.02)***	-0.08(+0.02)**	0.00(+0.01)	0.02(+0.01)*	0.01(+0.00)†
Icing (*β*_4_)	-0.04(+0.03)	-0.07(+0.04)	0.00(+0.02)	0.06(+0.01)***	0.05(+0.01)***
Pressure (*β*_5_)	-1.06(+0.15)***	-0.78(+0.20)***	0.02(+0.11)	0.10(+0.09)	0.00(+0.07)

Here

*** refers to “*p*-value” less than 0.001

** less than 0.01

* less than 0.05, and

† less than 0.1.

All reported cells were presented as coefficient (SE). SE: standard error.

**Fig 1:**
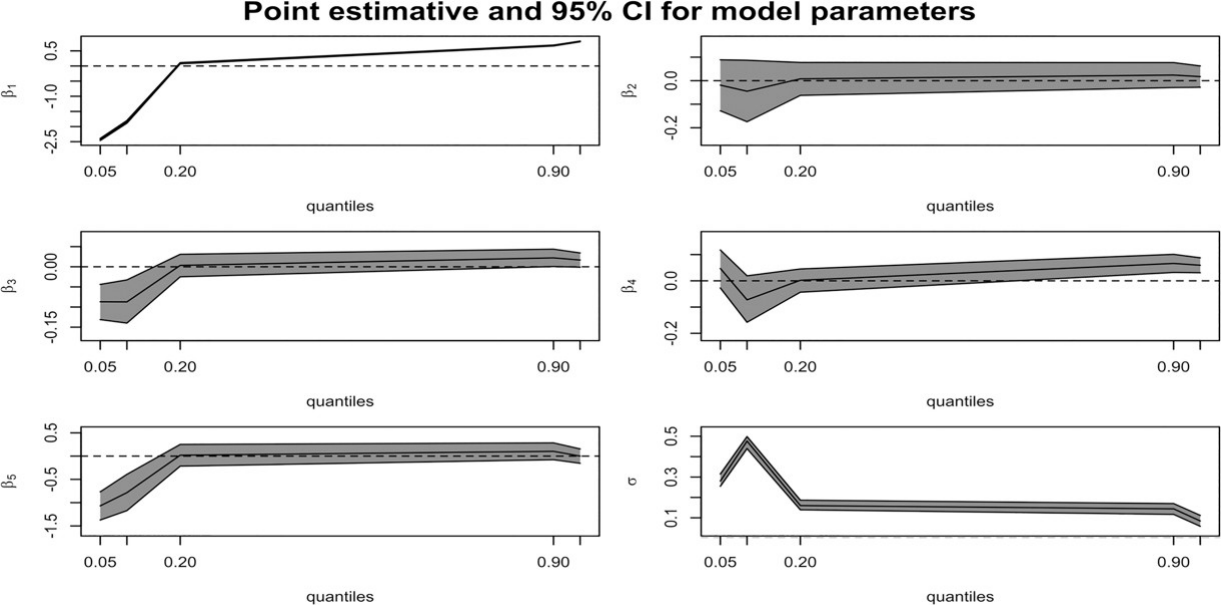
Estimated 95% CI for model parameters of field goals. Here, Intercept as (β_1_), Postseason as (β_2_), Away as (β_3_), Icing as (β_4_), and pressure as (β_5_).

**Fig 2:**
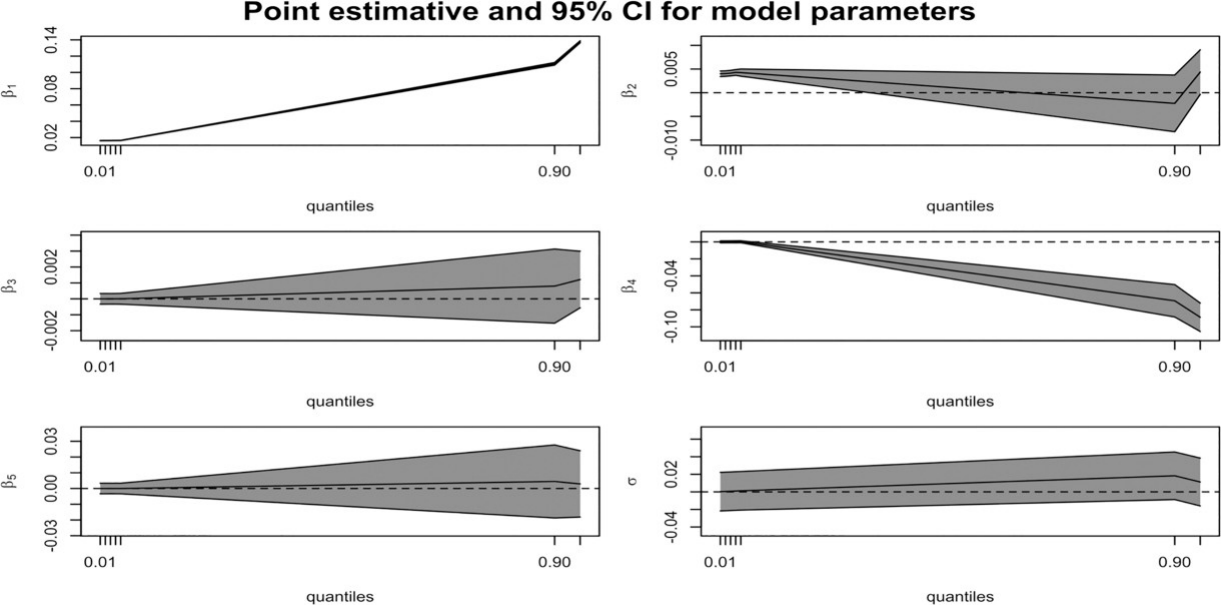
Estimated 95% CI for model parameters of extra points. Here, Intercept as (β_1_), Postseason as (β_2_), Away as (β_3_), Icing as (β_4_), and pressure as (β_5_).

As shown from Table 3 and β_2_ and β_3_ in Fig 1, a closer examination of the magnitude of the estimated coefficients reveals some similarities and dissimilarities among quantiles. First, only the factor of whether it was post-season or not (β_2_) in our model does not appear to influence the kicker’s true performance rate, since they are non-significant in nearly every quantile considered. With the exception of the area from the 0.2 quantile to the 0.9 quantile, there apparently exists a home field advantage for showing a negative impact for a kicker’s true performance rate (*p* < 0.05) of the visiting team, based on most missed shooting data below the 0.2 quantile. Figs 1 and 2 also gives the estimates (solid lines) and the 95% confidence bands (shaded gray areas) for the regression coefficients from different quantiles.

Second, as shown from Table 3 and β_4_ and β_5_ in Fig 1, icing and situational pressure are two of the most meaningful variables. The negative impact (*p* < 0.05) of situational pressure (β_5_) on kickers’ true performance begins to weaken till the 0.2 quantile, meaning that pressure can only impair kickers’ performance based on our results especially when happening in a game-winning or high-pressure situation without bad environmental conditions and long distances. This is reconfirmed by our further logistic quantile regression model in S3 Table using more categories of situational pressure (7 levels as the same in S1 Table as dummy variables from the highest to no effect), S3 Table shows the statistical evidence of missed FG attempts really lies in the higher pressure of competition (*p* < 0.05). The psychological aspect of situational pressure (especially the highest level) may be the choking factor not to be ignored but was not the sole factor contributing to the missed outcome of a FG kick. Our proof is obviously different from the conclusion to show only environmental influences in the previous study [32].

Besides, although icing is a common strategy used during the last moments of a close-ending game when the opposing head coach may ask for a timeout to an extended period of time possibly to contemplate negative outcomes, the kicker may fail to score below the 0.2 quantile (but *p* > 0.05); in contrast, it was found that icing or a timeout would increase the scoring probability while facing a worse environmental situation or longer kicking conditions over the 0.9 quantile (*p* < 0.05), and such performance is uncorrelated with a high-pressure situation (*p* > 0.05).

We observe from Table 4 and Fig 2 that the factors with significant impact on PAT include only whether the match is post-season, which is of significance from the 0.01 to the 0.05 quantile, while there is a decreasing trend with respect to icing or timeouts (β_4_) on scoring a PAT especially when facing a worse environmental situation over the 0.9 quantile (*p* < 0.05). The pressure kick effect of PAT is confirmed only by our further logistic quantile regression model in S4 Table using more categories of situational pressure (7 levels as the same in S2 Table as dummy variables from the highest to no effect), S4 Table shows the statistical evidence of missed PAT attempts lies in the highest-pressure level of competition from the 0.01 to the 0.02 quantile (*p* < 0.05), since missed PAT is a relatively rare event among kicks in the NFL 2000–2017.

**Table 4:** Logistic quantile regression model of extra point (2000–2017).

Variable	Quantile						
	0.01	0.02	0.03	0.04	0.05	0.9	0.95
Intercept (*β*_1_)	0.01(+0.00)***	0.01(+0.00)***	0.01(+0.00)***	0.01(+0.00)***	0.01(+0.00)***	0.11(+0.00)***	0.13(+0.00)***
Postseason (*β*_2_)	0.01(+0.00)***	0.01(+0.00)***	0.01(+0.00)***	0.01(+0.00)***	0.01(+0.00)***	-0.00(+0.00)	0.00(+0.00)†
Away (*β*_3_)	0.00(+0.00)	0.00(+0.00)	0.00(+0.00)	0.00(+0.00)	0.00(+0.00)	0.00(+0.00)	0.00(+0.00)
Icing (*β*_4_)	0.00(+0.00)	0.00(+0.00)	0.00(+0.00)	0.00(+0.00)	0.00(+0.00)	-0.06(+0.00)***	-0.08(+0.00)***
Pressure (*β*_5_)	0.00(+0.00)	0.00(+0.00)	0.00(+0.00)	0.00(+0.00)	0.00(+0.00)	0.00(+0.01)	0.00(+0.01)

Here

*** refers to “*p*-value” less than 0.001, ** less than 0.01, * less than 0.05, and

† less than 0.1.

All reported cells were presented as coefficient (SE). SE: standard error.

### Addressing unobserved heterogeneity of kicks with probability models

We report the maximum likelihood estimates of *u* and φ for the FG (segmented by field goal length similar to the way adopted by Morrison and Kalwani [27]) and PAT data from the 2000 to 2017 NFL seasons. Table 5 contains the results calculated by each three aggregated years, which are very compelling and supports the view that the kickers are getting better year after year, since *u* (average strength) for each distance group shows a statistically upward trend. Table 5 also displays the total number of FGs or PATs attempted in each distance group. For the kickers who kicked in 50-yards-or-more FGs during the 18 NFL seasons, the numbers of FGs attempted in each year group varied from 234 to 483, while the numbers of FGs attempted within 29 yards dropped from 851 to 785. The intervention effect of the new extra-point rule in 2015 was also shown in Table 5. It can be seen that the PAT attempted has been declining since 2015, while the total numbers of FGs attempted has been increasing. As Table 5 reveals, even in these aggregate data with a large sample size, the estimates of φ are very close to 0 for all kicks or kicks segmented by FG length. For example, they are all less than.036. These findings from aggregate data provide further support consistently for the inference of a lack of skill differences among these elite NFL kickers [27].

**Table 5:** Beta-binomial model.

Seasons	Distance (yard)	n	μ	φ
2000–2002	ALL	2957	0.772	0.006
	<29	851	0.943	0.010
	30–39	890	0.826	0.011
	40–49	982	0.642	0.000
	>50	234	0.526	0.000
	Extra point	3391	0.984	0.000
2003–2005	ALL	2905	0.797	0.009
	<29	868	0.958	0.021
	30–39	874	0.826	0.035
	40–49	879	0.703	0.013
	>50	284	0.531	0.003
	Extra point	3585	0.986	0.000
2006–2008	ALL	3024	0.826	0.007
	<29	901	0.964	0.000
	30–39	911	0.880	0.022
	40–49	911	0.742	0.000
	>50	301	0.533	0.035
	Extra point	3640	0.988	0.001
2009–2011	ALL	3015	0.820	0.000
	<29	921	0.962	0.011
	30–39	864	0.861	0.000
	40–49	868	0.734	0.000
	>50	362	0.566	0.001
	Extra point	3792	0.988	0.004
2012–2014	ALL	3117	0.848	0.004
	<29	783	0.974	0.000
	30–39	939	0.900	0.000
	40–49	936	0.799	0.010
	>50	459	0.630	0.000
	Extra point	3907	0.994	0.000
2015–2017	ALL	3148	0.847	0.000
	<29	785	0.972	0.000
	30–39	916	0.908	0.001
	40–49	964	0.776	0.018
	>50	483	0.644	0.007
	Extra point	3694	0.937	0.006

### To be skillful, or to be anti-stress

Finally, to show our analysis regarding beta regression models, we can further compare the central tendency and dispersion level of the differences in FGs of kickers during 2015–2017 as our example to see if they are related to any explanatory variable. The detail of this model is as shown in Formulas (6) and (7) from the section on materials and methods in this study. An extension of the beta regression model above which was employed by Simas et al. [38] is the variable dispersion beta regression model, meaning that in this model the precision parameter is not constant for all observations. Three explanatory variables are available: AP/Attempt, susceptibility to stress, and extremely great play. Definitions of all three variables are listed in Table 6 as well as in the section of materials and methods.

Table 6 shows the results of beta regression model for successful conversion of an FG. In model 1, the effects assessed were susceptibility to stress, and extremely great play. As model 1 reveals, there is heteroskedasticity of kicker performance that can be only captured by the regressor, susceptibility to stress (*p* < 0.001), which can also be interpreted as testing against the null hypothesis of equidispersion among kickers. On the other hand, extremely great play, an idea similar to using kickers’ raw make percentages for long kicks or difficult kicks can only explain the mean performance of different kickers.

**Table 6:** Beta regression model.

	Variable	Model 1	Model 2
Mean model with logit link	Intercept	1.25(0.18)***	-0.31(0.04)***
	AP/Attempt	-	2.52(0.08)***
	Susceptibility to stress	0.10(0.16)	-0.12(0.05)*
	Extremely great play	0.45(0.17) **	-0.03(0.04)
Precision model with log link	Intercept	2.18(0.35)***	6.82(0.73)***
	AP/Attempt	-	-3.28(1.05)**
	Susceptibility to stress	1.55 (0.35) ***	1.72(0.37)***
	Extremely great play	-0.18 (0.51)	-0.07(0.58)
**AIC**		**-105.5895**	**-215.4941**

Here

*** refers to “*p*-value” less than 0.001

** less than 0.01

* less than 0.05, and † less than 0.1.

All reported cells were presented as coefficient (SE). SE: standard error.

***AP/Attempt*** here is slightly different to the definition of the numerator by Clark et al. [32], since we use the sum of residuals (additional points, AP) for one kicker instead of the sum of (3*residuals). The sum of residuals in a high-pressure situation over attempts is ***susceptibility to stress*** of one kicker (using the cut-off point of a higher-pressure condition in S1 Table, not original high-pressure condition in Clark et al. [32]). ***Extremely great play*** by one kicker represents the sum of one kicker’s residuals under model’s predicted likelihood of success (e.g., less than 20 percentile from all the population, *P*_20_ = 0.72) divided by counts of these attempts.

Model 2 was subsequently employed to explore whether the source of the whole skill measurement, AP/Attempt, made a difference in the successful outcome of FG kicks. Thus, it appears that the adding variable, AP/Attempt, did play the major explanatory role as the improvement of AIC value shows, because if this was the case in both central tendency and dispersion model, extremely great play (*p* > 0.1), should have been influenced by the appearance of AP/Attempt in the model. In particular, as model 2 reveals, we can express this result as this is evidenced in our beta regression models where the main performance-discriminating factor is not only skill of kickers but also susceptibility to stress.

## Discussion

Sports analytics have usually focused on the study of choking in free throws on the court in basketball, because each free throw attempt is an uncontested shot taken from the same distance and location without weather influences from the outdoor environment. We have exploited statistical modeling approaches to extracting the situational effect in natural-field-setting contexts generating many fruitful observations from the broader perspective on pressure kicks in the NFL 2000–2017, associated with or without adjusting for the difficulty of kicks given the specific environmental and distance conditions. On the other hand, many researchers often omit the role of residuals, the random components in recognition that other factors are not included in the regression models, but we instead derived them effectively as various measurable indicators such as true performance, extremely great play, and susceptibility to stress of kickers. We have large bodies of work on analysis of residuals that may replace some hard-to-collect or poorly measured, observed explanatory factors in original big data. Our findings showed that the psychological/situational variables could play a more important role in pressure kicks. What is more, our statistical evidences on NFL kickers could further support not only the “distraction hypothesis” but the “automatic execution hypothesis” outlined in our literature review. Accounting for attempt difficulty allows us to better understand and investigate the kickers’ true performance.

This paper was originally intended as a reply to the study “Going for Three: Predicting the Likelihood of Field Goal Success with Logistic Regression” [32]. We know from traditional logistic regression that factors impacting FG% are often natural ones such as distance and environment, while situational pressure does not show any significant impact in Clark et al. [32]. We checked the results reported in their paper and found noticeably different significant variables in our study according to sampling from different years, yet the estimates in FG models would still indicate the unpresented situational pressure effect as reported by Clark et al. An extended analysis of 7-category pressure reveals that pressure kicks are mostly consistent with the two parts of data divided by higher-pressure condition in 2000–2017 (in S1 Table, not original cut point of high-pressure condition in Clark et al. [32]) and would be explored by logistic quantile regression for addressing rare-event problems in NFL kicks, an alternative statistical procedure to weight the data to select on the dependent variable. Hence, we found the existence of a situational pressure effect and conquered challenges from the original dataset in which there are inherent risks from out-field and a high proportion of makes in FG and PAT. Our various statistical modeling designs checked potential endogenous selection bias, especially through an estimation strategy of different subsample analysis to classify and test more accurately the influence from pressure (Table 1, S3 and S4 Tables). In particular, as we show in the research results, the misclassification of pressure levels may result in no statistical evidence to claim that worse performance under pressure kick in the NFL is persistent. We utilize a “pressure” classification system (S1 and S2 Tables), and compare and contrast other variables between our study and an earlier analysis [32], but the researchers should keep in mind that it is generally not viewed favorably as to make continuous variables categorical and may result in a loss of power [39] and misclassification.

In summary, since most close score differences of games in the NFL involve at least a few opportunities to attempt an offensive strategy for kicks in the final minutes or overtime, it seems highly plausible that psychological/situational variables may show a combined reaction to affect pressure kicks as we have learned. Furthermore, we were writing this paper around January 2019, and seeing the Chicago Bears kicker 'choke' and cost them the wild card game is another vivid example of the phenomenon occurring again in the NFL playoffs. A major strength of this study is the availability of comprehensive information providing an in-depth evaluation of performance under the pressure of competition, using a renowned play-by-play and long-range database from the NFL, and depicting a clearer picture of the kickers’ skill and performance through all full games rather than only selected game situations during the final minutes, in order to capture the change process in the performance of athletes at normal times and critical moments.

In addition, this study was able to address the problem of heterogeneity among players in most studies conducted in sports outcome research. Heterogeneity in the behaviors of individuals became a core premise upon which any game strategy was based, and the probability theory could help enable managers or scholars to identify how to select kickers from our results. We propose a beta-binomial model of individual-level behavior which is “summed” across individuals to obtain a model of aggregate behavior. We further view the parameters of probability distribution as individual-level latent characteristics. In particular, incorporating covariates with probability distribution models such as the beta regression model above was potentially generalized to accommodate a wide range of analyses of latent characteristics to describe/predict behavior using not only proximity of observed variables but also derived novel ones as shown. Future research may try field experiments such as using assisted wearable biosensor devices [40] to look deeper into the other hidden aspects of a kicker under pressure and how the outcome of a pressure kick varies due to the individual player’s mental state. Further research is also needed in Sports analytics, in particular, given that players play different numbers of games, a multilevel model or hierarchical model could be considered.

Finally, our findings showed that when the kickers did a PAT like a 33-yard FG attempt after the PAT rule change, the environmental and longer distance impacts for the players were heightened, causing the PAT conversion rate to drop. Furthermore, we should indicate some limitations and remarks in relation to the research data and process. For instance, all the records of environmental conditions relate to kickoff of that game day and are not specific to the time of each kick, alternative categorization of variables or rare-event data issues in model building may show a non-negligible impact on the value and significance of the coefficient, and a timeout called by either head coach was considered “icing the kicker.” All timeout called by either head coach was considered “icing the kicker,” which is the same way by which Clark et al. [32] analyzed the icing strategy Categorizing “icing” as either timeout called by an opposing coach (timeout mostly by the rivals), or timeout called by one’s own coach had a negative effect as well, but not significant. Interestingly, it was found that timeout strategy increased scoring probability, especially when facing a worse environmental situation combined with longer kicking conditions, irrespective of whether it was a pressure kick or not. This can be accounted by kickers’ self-reports. For example, Lawrence Tynes, a kicker for the New York Giants, in 2008 interview with USA Today, said: “Perfect. Coaches are going to learn not to do that icing. Basically, you get a chance to clean up a spot, get a good look at the goal posts, look at the wind, smell the air and let my caddie tell me I’m going to drill it. You’re almost more anxious without a timeout. Then, when they give you a timeout, you get to take a couple of deep breaths [7].” In short, pressure and difficulty, when it comes to extended time before the kick, may be operating differently and in an opposing manner.

The research also suggests that players who are able to maintain their performance under circumstances of change do so because of aspects of both physiology and psychology, and this is evidenced in the case of our beta regression models where the main performance-discriminating factor is not only the skill of kickers but also their susceptibility to stress. Our findings show that NFL teams can differentiate their kickers’ performance variations in terms of their performance during pressure kicks over using kickers’ performance for longer kicks or difficult kicks, especially in relation to considering the possible sampling selection bias from some kickers who have suffered more times in worse environmental conditions. Above all, we concluded that with better skills and tough mental states together a kicker can burst on the scene and find fame in his promising future, and he may very well become indispensable for his team. From our complete statistical investigation of pressure kicks, we can better clarify the role ambiguity of many observed environmental and situational factors, and unobserved or latent characteristics discussed in past literature.

## Supporting information

S1 Table Categorization of situational pressure when field goal. (PDF)

S2 Table Categorization of situational pressure when point after touchdown. (PDF)

S3 Table Logistic quantile regression model of field goal with 7-category pressure. (PDF)

S4 Table Logistic quantile regression model of extra point with 7-category pressure. (PDF)

S1 Fig Nonlinear relationship with field goal percentage under different types of precipitation. (TIFF)

S2 Fig Nonlinear relationship with field goal percentage under different types of turf. (TIFF)

S3 Fig Nonlinear relationship with field goal percentage under different types of temperature. (TIFF)
