# Supplementary material for: Choking under the pressure of competition: A complete statistical investigation of pressure kicks in the NFL, 2000–2017
Source: PLoS One. 2019 Apr 2;14(4):e0214096. doi: 10.1371/journal.pone.0214096 (PMC6445473; doi:10.1371/journal.pone.0214096)
Supplement: S1 Table — (PDF) [file pone.0214096.s001.pdf]

S1. Categorization of situational pressure when field goal.

Time remaining	Score Differential	Effect of Kick	7-category Pressure	2-category Pressure
4th quarter	> |21|	No effect	No	Low
4th quarter, < 2 minutes	> |8|	No effect	No	Low
4th quarter, < 2 minutes	< −7	No effect	No	Low
1st-3rd quarters	Any	Regular effect	Low	Low
4th quarter, > 2 minutes	< |21|	"Close" 4th quarter	Medium	Low
4th quarter, < 2 minutes	5, 6, 7, 8	Helps seal game	Medium	Low
4th quarter, < 2 minutes	-4, -5, -6	Come within 3	Medium	Low
4th quarter, < 2 minutes	4	Opponent needs TD	Medium-high	High
4th quarter, < 2 minutes	3	Opponent needs TD	Medium-high	High
4th quarter, < 2 minutes	1, 2	Opponent needs TD	High	High
4th quarter, < 2 minutes	0	Win. If miss, OT	Higher	High
Overtime	0 (Any)	Win. If miss, more OT	Higher	High
4th quarter, < 2 minutes	-3	OT. If miss, lose	Highest	High
4th quarter, < 2 minutes	-1, -2	OT. If miss, lose	Highest	High

For Table2

For Table3,5

Source : (Clark, Johnson, & Stimpson, 2013)
