# Supplementary material for: Choking under the pressure of competition: A complete statistical investigation of pressure kicks in the NFL, 2000–2017
Source: PLoS One. 2019 Apr 2;14(4):e0214096. doi: 10.1371/journal.pone.0214096 (PMC6445473; doi:10.1371/journal.pone.0214096)
Supplement: S2 Table — (PDF) [file pone.0214096.s002.pdf]

S2. Categorization of situational pressure when point after touchdown.

Time remaining	Score Differential	Effect of Kick	7-category Pressure
4th quarter	$> \lvert 21 \rvert$	No effect	0
4th quarter, < 2 minutes	$> \lvert 8 \rvert, 8$	No effect	0
1st-3rd quarters	Any	Regular effect	1
4th quarter, > 2 minutes	$< \lvert 21 \rvert$	"Close" 4th quarter	1
4th quarter, < 2 minutes	4,5,6	Regular effect	1
4th quarter, < 2 minutes	1,2	Regular effect	1
4th quarter, < 2 minutes	-2,-3	Regular effect	1
4th quarter, < 2 minutes	-5,-6,-7	Regular effect	1
4th quarter, < 2 minutes	7	Helps seal game	2
4th quarter, < 2 minutes	3	Opponents need TD	3
4th quarter, < 2 minutes	-8	Come within 7	3
4th quarter, < 2 minutes	-4	Come within 3	4
4th quarter, < 2 minutes	0	Win. If miss, OT	5
4th quarter, < 2 minutes	-1	OT. If miss, lose	6

For Table3
