# Supplementary material for: Choking under the pressure of competition: A complete statistical investigation of pressure kicks in the NFL, 2000–2017
Source: PLoS One. 2019 Apr 2;14(4):e0214096. doi: 10.1371/journal.pone.0214096 (PMC6445473; doi:10.1371/journal.pone.0214096)
Supplement: S3 Table — (PDF) [file pone.0214096.s003.pdf]

S3. Logistic quantile regression model of field goal with 7-category pressure.

Variable	Quantile				
	0.05	0.1	0.2	0.9	0.95
Beta 1	-2.27(+0.04)***	-1.80(+0.05)***	0.09(+0.02)***	0.65(+0.02)***	0.81(+0.01)***
Beta 2	-0.01(+0.05)	-0.05(+0.06)	0.00(+0.03)	0.02(+0.02)	0.01(+0.02)
Beta 3	-0.08(+0.02)***	-0.08(+0.02)**	0.00(+0.01)	0.02(+0.01).	0.01(+0.00)
Beta 4	-0.03(+0.03)	-0.08(+0.04) †	0.00(+0.02)	0.06(+0.01)***	0.05(+0.01)***
Beta 5	-0.17(+0.04)***	-0.05(+0.05)	0.00(+0.02)	0.01(+0.02)	0.00(+0.01)
Beta 6	-0.19(+0.05)***	-0.01(+0.06)	0.00(+0.03)	0.01(+0.02)	-0.01±0.01)
Beta 7	-0.47(+0.18)*	-0.46(+0.21)*	-0.00(+0.15)	-0.08(+0.10)	0.03(+0.08)
Beta 8	-0.04(+0.40)	0.04(+0.35)	0.03(+0.14)	0.20(+0.14)	0.12(+0.16)
Beta 9	-1.20(+0.31)***	-0.80(+0.35)*	0.01(+0.23)	0.14(+0.16)	0.02(+0.14)
Beta 10	-0.77(+0.22)***	-0.84(+0.24)***	0.02(+0.13)	0.08(+0.10)	-0.01(+0.12)

*** refers to "p-value" less than 0.001, ** less than 0.01, * less than 0.05, and † less than 0.1. All reported cells were presented as coefficient (SE). SE: standard error.

Here, *Intercept* as (Beta 1), *Postseason* as (Beta 2), *Away* as (Beta 3), *Icing* as (Beta 4), and *Pressure levels* as (Beta5-10).
