# Supplementary material for: Choking under the pressure of competition: A complete statistical investigation of pressure kicks in the NFL, 2000–2017
Source: PLoS One. 2019 Apr 2;14(4):e0214096. doi: 10.1371/journal.pone.0214096 (PMC6445473; doi:10.1371/journal.pone.0214096)
Supplement: S4 Table — (PDF) [file pone.0214096.s004.pdf]

S4. Logistic quantile regression model of extra point with 7-category pressure.

Variable	Quantile						
	0.01	0.02	0.03	0.04	0.05	0.9	0.95
Beta 1	0.01(+0.00)***	0.01(+0.00)***	0.01(+0.00)***	0.01(+0.00)***	0.01(+0.00)***	0.11(+0.00)***	0.13(+0.00)***
Beta 2	0.01(+0.00)***	0.01(+0.00)***	0.01(+0.00)***	0.01(+0.00)***	0.01(+0.00)***	-0.00(+0.00)	0.00(+0.00) †
Beta 3	0.00(+0.00)	0.00(+0.00)	0.00(+0.00)	0.00(+0.00)	0.00(+0.00)	0.00(+0.00)	0.00(+0.00)
Beta 4	0.00(+0.00)	0.00(+0.00)	0.00(+0.00)	0.00(+0.00)	0.00(+0.00)	-0.06(+0.00)***	-0.08(+0.00)***
Beta 5	0.00(+0.00)	0.00(+0.00)	0.00(+0.00)	0.00(+0.00)	0.00(+0.00)	0.00(+0.00)	-0.00(+0.00)
Beta 6	0.00(+0.00)	-0.00(+0.00)	-0.00(+0.00)	-0.00(+0.00)	-0.00(+0.00)	0.00(+0.01)	0.00(+0.01)
Beta 7	0.00(+0.00) †	0.00(+0.00) †	0.00(+0.00) †	0.00(+0.00)	0.00(+0.00) †	0.02(+0.01)	0.06(+0.01)***
Beta 8	0.00(+0.00)	0.00(+0.00)	0.00(+0.00)	0.00(+0.00)	0.00(+0.00)	-0.07(+0.14)	-0.10(+0.19)
Beta 9	0.00(+0.00)	0.00(+0.00)	-0.00(+0.00)	-0.00(+0.00)	-0.00(+0.00)	0.02(+0.01) †	0.00(+0.01)
Beta 10	-4.99(+0.44)***	-4.99(+0.44)***	-0.00(+0.00)	-0.00(+0.00)	-0.00(+0.00)	0.00(+0.02)	-0.02(+0.03)

*** refers to "p-value" less than 0.001, ** less than 0.01, * less than 0.05, and † less than 0.1. All reported cells were presented as coefficient (SE). SE: standard error.

Here, *Intercept* as (Beta 1), *Postseason* as (Beta 2), *Away* as (Beta 3), *Icing* as (Beta 4), and *Pressure levels* as (Beta5-10).
