# Supplementary figures and images for: Choking under the pressure of competition: A complete statistical investigation of pressure kicks in the NFL, 2000–2017
Source: PLoS One. 2019 Apr 2;14(4):e0214096. doi: 10.1371/journal.pone.0214096 (PMC6445473; doi:10.1371/journal.pone.0214096)

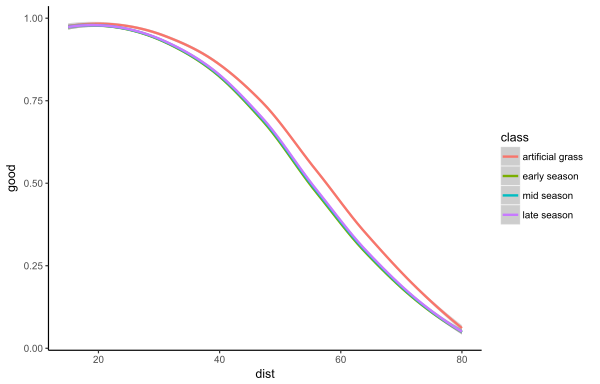

Supplement: S1 Fig — (TIFF) [file pone.0214096.s005.tiff]

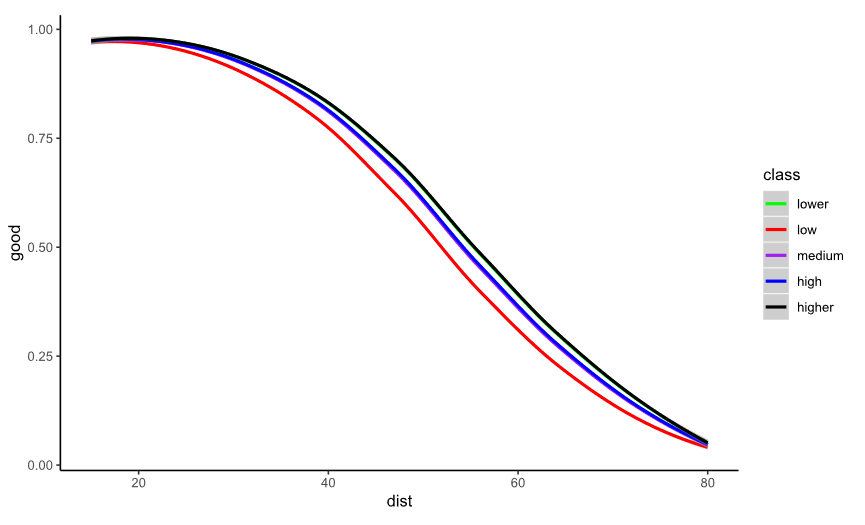

Supplement: S2 Fig — (TIFF) [file pone.0214096.s006.tiff]

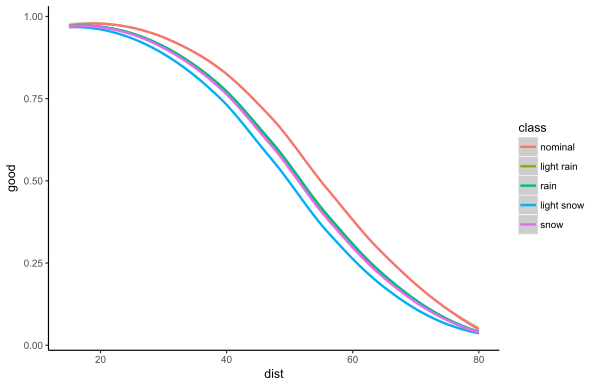

Supplement: S3 Fig — (TIFF) [file pone.0214096.s007.tiff]
